# Transforming trash to treasure Cultural ambiguity in foetal cell research

**DOI:** 10.1186/s13010-021-00104-y

**Published:** 2021-09-15

**Authors:** Andréa Wiszmeg, Susanne Lundin, Åsa Mäkitalo, Håkan Widner, Kristofer Hansson

**Affiliations:** 1 Department of Arts and Cultural Sciences, The Joint Faculties of Humanities and Theology, Lund University, LUX, Helgonavägen 3, 221 00 Lund, Sweden; 2 Department of Public Health, University of Copenhagen, Medical Museion, Fredericagæde 18, 1310 København K, Denmark; 3 Department of Social Work, Faculty of Health and Society, Malmö University, Citadellsvägen 7, 205 06 Malmö, Sweden; 4 Stellenbosch Institute of Advanced Study (STIAS), Wallenberg Research Centre At Stellenbosch University, Marais Road, Stellenbosch, 7600 South Africa; 5 Department of Education, Communication and Learning, University of Gothenburg, Läroverksgatan 15, 411 20 Gothenburg, Sweden; 6 Faculty of Medicine, Lund University, SUS EA-Block, Getingevägen 4, 221 00 Lund, Sweden

**Keywords:** Foetal cells, Embryos, Abortion, Transplantation, Pollution behaviour, Ritual, Foetal waste, Abject, Embryonic ambiguity

## Abstract

**Background:**

Rich in different kind of potent cells, embryos are used in modern regenerative medicine and research. Neurobiologists today are pushing the boundaries for what can be done with embryos existing in the transitory margins of medicine. Therefore, there is a growing need to develop conceptual frameworks for interpreting the transformative cultural, biological and technical processes involving these aborted, donated and marginal embryos. This article is a contribution to this development of frameworks.

**Methods:**

This article examines different emotional, cognitive and discursive strategies used by neurobiologists in a foetal cell transplantation trial in Parkinson’s disease research, using cells harvested from aborted embryos. Two interviews were analysed in the light of former observations in the processing laboratories, using the anthropologist Mary Douglas’s concept of pollution behaviour and the linguist, philosopher, psychoanalyst and feminist Julia Kristeva’s concept of the abjective to explain and make sense of the findings.

**Results:**

The findings indicate that the labour performed by the researchers in the trial work involves transforming the foetal material practically, as well as culturally, from trash to treasure. The transformation process contains different phases, and in the interview material we observed that the foetal material or cells were considered objects, subjects or rejected as abject by the researchers handling them, depending on what phase of process or practice they referred to or had experience of. As demonstrated in the analysis, it is the human origin of the cell that makes it abjective and activates pollution discourse, when the researchers talk of their practice.

**Conclusions:**

The marginal and ambiguous status of the embryo that emerges in the accounts turns the scientists handling foetal cells into liminal characters in modern medicine. Focusing on how practical as well as emotional and cultural strategies and rationalizations of the researchers emerge in interview accounts, this study adds insights on the rationale of practically procuring, transforming and utilizing the foetal material to the already existing studies focused on the donations. We also discuss why the use and refinement of a tissue, around which there is practical consensus but cultural ambiguity, deserves further investigation.

## Background

Besides being a source of potent cells in regenerative medicine, the embryo is a product of the transformative states of pregnancy, birth giving and abortion, and is consequently associated with the margins of life and death. Objects associated with these borderlands are often culturally considered holy as well as threatening. Embryos are therefore symbols – or icons – of life itself [1] of vitality [2] and potentiality [3] as well as of innocence and of sin—as this paper will demonstrate. In Parkinson’s disease research, which is the case study for this paper, cells are harvested from aborted embryos and processed into cell suspension to be used for transplantation in a medical trial. So while the foetal cells^1^ are, in one way, isolated from the outside world, they still have connections to the surrounding society in many ways. Their origin extend to the practical work (see e.g. [4–6])that is done in the clinic – to the wombs and minds of aborting women and their families – through the different facilities handling the foetal material in different stages and the hands of midwives and researchers mobilizing and refining it, as well as into the minds and brains of future rats and patients, and into the hopes and dreams of the media and the afflicted [7]. There, it highlights the same issues as Douglas’s ‘concept of pollution behaviour, namely, that the foetal cells are something that can be both specific and formless; that they can create order or be a disorder; that they may give life, while a potential life is ended [8]. Therefore, it poses conceptual problems for the researchers dealing with it practically on daily basis in the laboratories as well as in relation to the outside world.

In this article, Douglas’s [8] concept of pollution behaviour is suggested as a key concept to understand the cultural processes of turning the marginal, aborted, abject [9] embryo, from trash to treasure in a biomedical setting. Briefly, the core of Douglas’s concept can be described as the practice of cleaning up cultural ‘dirt’ within a community, that displacement of a specific object within it, renders. This is done in many different ways depending on the context and the type of pollution, but it all serves the same purpose, which is to neutralize a threat and to reinstate communal social and cultural order.

With the concept of pollution behaviour, we want to examine different strategies used by – and expressed in accounts of – neurobiologists to handle cognitive and emotional challenges emerging from the processing of aborted tissue in a foetal cell transplantation trial. We do this by connecting interview accounts focusing on their professional practice to previous observations of it. The article offers a cultural perspective on how foetal cells, as scientific artefacts of foetal origin, can be considered marginal objects of waste, both culturally dangerous and simultaneously powerful. Earlier studies have raised similar questions. Ariss [10] as well as e.g. Waldy [11], have conceptualized foetal materials as cultural waste. Pfeffer [12] and Kent [13] have mapped cultural and emotional motivations for foetal donation for stem cell research, among possible donating women in Great Britain as well as among different practitioners facilitating donation. Focusing on practical as well as cultural strategies and rationalizations of the researchers receiving and processing the tissue, this study adds insights on the rationale of practically procuring, transforming and utilizing the foetal material, thus supplementing previous studies focused on the donations.

Responding to the issues that the researchers face through the framework of pollution behaviour, we hope to offer the researchers an explanatory model, and also to give meaning outside the biomedical paradigm. One such example is the use of foetal cells derived from aborted embryos in research and in clinical trials on neurological disorders. The neurobiologists are recurrently reminded of the origins of the foetal material in their daily work in the laboratory. Its double status as an aborted, private kind of waste, as well as an available source of vital, valuable regenerative cells makes it ambiguous (cf. [14]) – and gives it what will here be described as ‘embryonic ambiguity.’ Different tasks and stages of the cell refinement process either recall or obscure the relation of the cells to the aborted embryo and to the donating woman. As these cells are also scarce, their regenerative value makes them a rare commodity [15, 16]. The number of abortions performed in the near region does not cover the required amount needed to secure enough cells for the patients, and embryos must be transported between the cooperating centres in the trial to try and cover each other’s needs. Different strategies and attitudes are needed and developed in the cell laboratory and the animal transplantation laboratory, to deal with the relation between the practical cell refinement process and the more figurative and symbolic cleansing process, as well as with the scarcity of cells – in short; with the ‘embryonic ambiguity’. It is not always a smooth and unproblematic procedure. In these frictional events, the development of such strategies is highly visible. Therefore they make up a methodologically fruitful arena of examination.

This study follows neurobiologists in their work in a transplantation trial, in which they employ the regenerative potential of aborted embryos. They harvest dopaminergic brain cells from aborted embryos, which are used in transplants called cell suspensions, to patients in the early stages of development of Parkinson’s disease. These cells have the ability to alleviate and possibly reverse symptoms of the disease, by partly restoring the patient’s lacking dopamine production. Showing a high success rate in using foetal cells to a larger number of Parkinson patients is of very large significance to the research team and to the trial, as well as to the broader field of cell transplantation research as a whole.

## Methods

The two interviews in focus for this article are contextualized and supported by a body of ethnographic materials, produced during observations in the cell and animal laboratories, as well as participation in the regional planning meetings of the trial during two years. The observational and fieldwork notes amount to 215 handwritten pages in total, of which about 70 pages consists of laboratory observations. Approximately 30 h was spent on observations. There is a smaller photographic documentation of the processing of cell suspension. The material also includes 25 documents; official as well as so-called grey documents.^2^ They range from ethical permits to descriptions of good clinical practice and standard operating procedure. Still, the analyzed research material for this article is arguably not very large, as the main focus are two semi-structured interviews.

In exploring an issue of general cultural importance and applicability, however, the aim is not to achieve generalizability of the results. Rather, we want to show that it is a fruitful as well as highly important arena for further research, and suggest it is given further scholarly attention. By accounting for what we argue are some expressions of the mechanisms of pollution behaviour, the article gives higher transparency to an activity that is opaque to many outside the biomedical field. Moreover, as it is often surrounded by biomedical parlance and terminology, the cultural side of ethics goes unexplored. This is unfortunate, as such issues are of great societal interest and importance.

The empirical basis for this article is the two semi-structured interviews with researchers in cell- and animal transplantation laboratories respectively, as the accounts of these researchers relate and resonate well with the two foundational theories used for analysis. The basis for the dissertation project as a whole – which this article is part of – is ethnographic research methods. These set of methods and the data rendered by e.g. observations and document analysis also works as an informative back drop to this article, as it is central for studying the activities of the laboratories [4–6]. Ethnographic methods are well adapted to this kind of study, where the aim is to reach and visualize mundane and vague social and cultural processes. The point of departure of these methods is for the researcher to “be there”, and in doing so, reach into and problematize the practices in the situations in which they occur [17]. These practices are not always verbalized in the setting, but may be lifted and discussed in interviews with the participants at a later point – which is the method used for creating the here analysed interview material. This allows the researcher in the laboratory to reflect upon the events and thereby also broaden as well as deepen the common understanding of the events [18]. The material described above forms the basis for the argumentation in this article, using the two interviews as main means for visualizing and verbalizing the findings and the analyses of them.

The interviews were conducted following upon repeated observations of the daily tasks of the researchers within the cell and animal laboratories of the trial. The aim of the observations was to see how the materiality of the trial – such as equipment, machines and foetal tissue – was made to come into correspondence with the conceptual instructions and regulations of the documents, through the manual skills of the researchers in the laboratories when manufacturing foetal cell suspension. The interview accounts are focused on the researchers’ professional practice, and have been preceded by observations of these same practices, and by a study of the documents guiding the process. The materials are then connected to each other, in order to create a fuller picture of the scientific practice, materially as well as conceptually.

The interview guides were designed for discussing and elaborating on the findings from the observations, and focused on the researchers’ perception of the relation between the conceptual and the material when producing the cell suspension. The result was two extensive semi-open-ended interviews lasting 1.5–2 h each, with two junior neurobiologists in their thirties, given the aliases James and Emma for reasons of confidentiality. The interviews invited elaboration together with each of them, on different cultural and emotional meaning and strategies that the foetal cells enact with them in their daily work in the trial. As the interviews connect philosophical and moral issues with practical laboratory tasks and work experience of the researchers, they offer an understanding of how the materiality of the embryos sometimes works together with, and sometimes against, their professional skills in practice – now and then creating tensions in the processing of foetal cell suspension. In the interviews, the researchers gave accounts of their work in the laboratory.

The present article reports on the discursive strategies they employ to talk about the process of relating cognitively and emotionally to the foetal material they handle in their daily work routine. Their experience of friction in interaction and in communication in the laboratory work between staff of similar, as well as different, professions is explored. Their reactions and responses in the interviews and their argumentation around their professional handling of foetal cells are of course personal and non-generalizable. The crucial point here, however, is the way in which their personal strategies function to mediate between the standardizations of the laboratories and the medical trial conditions and contexts that are addressed in the regulating documents which are also part of the empirical material – and a broader social as well as cultural landscape in which the research, as well as the researchers themselves, are situated. The scripts from the interviews form the empirical basis for this article, and the quotations discussed in the Results section are excerpts from there.

Wiszmeg constructed the ethnographic material as part of a broader dissertation project, and Hansson and Lundin have supervised her in this process. Wiszmeg performed the first step in the analytical process, where the interview accounts were thematized by ethnographic content analysis [19]. In the next step, Hansson and Lundin read these themes carefully numerous times and looked for biases. In step three, Wiszmeg theorized the themes and translated them into categorizations and conclusions. In this stage Mäkitalo has been connected to the analysis work to obtain a fourth opinion and for an analysis round on the overall conclusions. Widner is medically responsible for the trial studied here and has provided the medical competence for this article, by reading it through and correcting the analysis according to medical standards and terminology.

## Disposition

First, the cultural role of dirt, waste and the management of it by pollution behaviour will be presented as conceptualized by Douglas [8]. These concepts will thereafter be elaborated on to specifically discuss foetal material as an abjective [9] waste product [10, 12]. We discuss how the material is made usable and thus reintegrated to culture and society by pollution behaviour. Next, the neurobiologists and their work setting will be briefly presented ethnographically, as an introduction to the analytical part of the article. Following upon that, the interviews will be discussed thematically, using the concept of pollution behaviour as an analytical model. Finally, the implications of the findings will be elaborated upon in relation to modern developments of regenerative medicine at large. We discuss why expressions of ambiguity and discomfort in the interface of private versus professional aspects of the researcher as person should be examined at the borders of science(s) and society.

## Theory

### Dirt and pollution behaviour

Douglas’s book *Purity and Danger* was written as an attempt to create a general and systemic theory of ritual cleanness in religious or spiritual ritual. As such it is far from today’s high-tech laboratories, but as we shall see in the following it is highly relevant for gaining an understanding of the cultural and societal function of the laboratory processing of foetal tissue into cell suspension as a cultural phenomenon. It is largely due to the cultural mechanism transforming aborted embryos from waste into resource that they are possible to utilize as a resource for cells in regenerative medicine.

To reach a more general understanding of the procedure as a form of ritual cleanness, we need to look at the opposite: ritual pollution and the role of so-called pollution behaviour in making what is considered dirty clean again. To understand the dialectics of pollution behaviour it is essential to understand that the function of the taboo [8] is to protect local consensus as well as to confront the culturally ambiguous in a community ([20], p.11). Anything that challenges social and cultural classifications and patterns within this system activates so-called pollution behaviour, which excludes the specific matter or object from this order as being dirt or waste [8]. Dirt is basically and generally, in Douglas’s account, ‘matter out of place’ ([20], p. 44, 50). There are indeed designated places where different matter should and should not be located, and the categorization work behind that notion is itself a sign of cultural activity. When pollution has occurred by displacement of such an object, the community needs to clean it up. The modus operandi or ritual used for doing this, of course differs between communities and depending on the nature of pollution.

Applying Douglas’s concept of pollution behaviour [8] to the use of foetal material in regenerative medicine illuminates many of its processes. The first one is the making of aborted embryos or foetuses into waste, and a source that is possible to utilize for societal purposes generally conceived of as good. Labelling the aborted foetus as waste makes the foetal material available for research. The action of utilizing the resource has been legitimized with the argument that it is the economical and responsible thing to do in a medical economy with scarce resources of biological tissues. Not using it then would in itself be an act of wasting [8, 10, 11, 21]. Labelling the foetus waste makes it profane, worldly. To bring meaning to the discarded potential for life represented by foetal cells and reinstate them into social and cultural order, they need to be refashioned so that their vitality may be harnessed for other positive purposes. They need to be transformed from *trash to treasure*. They can now be manipulated, made of use for worldly purposes and commodified. According to Douglas’ pollution theory, the harvesting and use of foetal material is therefore easily defendable since its holy status has already been compromised by abortion. Reinstating communal and social order by using it is then seen as the right and rational thing to do, since it offers people healing and redemption.

### Donations—gifts and waste

Donations of tissues, cells and organs in medicine are most often considered gifts [10]. When a gift is given, it is the recipient’s to care for and to use within the limits of a social and cultural contract. The gift-giver or donor has given up the right to influence subsequent events concerning the gift or donation, and is not supposed to expect benefits from the donation. At least, this is the idea of altruistic donation, which is prevalent in a Northwestern setting of medicine and care [10]. Still, a reciprocal relationship in donation is common for other kinds of reproductive tissues or products, such as IVF embryos considered leftovers [12], or cord blood donation [22]. Interestingly enough, no obvious mutual benefit can be discerned in the case of donating aborted foetal tissue, even if it has been a widely accepted explanation that it may assist the aborting women in a possible grieving process [12]. What complicates the donation of aborted foetal material is that, while it is the aborting woman’s right (in Swedish law) to decide on its use or disposal – the foetus in itself has had a theoretical chance of becoming an autonomous individual, a biographical person. Still, it has no legal status in itself, but is considered a tissue part of the woman’s body and therefore treated as a tissue donation^3^ [12].

In debates concerning abortion and experimental or therapeutic medical use of foetal material, questions arise as to whether a foetus is yours to give as an aborting woman. In contemporary Sweden, it belongs to a pregnant/aborting woman judicially (and practically), but is still seen as an organically vital entity on its own even if it cannot survive unaided outside the uterus. The potentiality of life and personhood in foetuses and embryos still trigger unease, insecurity and cultural, social and philosophical ambivalence, no matter what national law and jurisdiction dictate. One way of understanding the ambivalence that an aborted embryo enacts is that abortions – spontaneous or intentional – generally are considered a wasted pregnancy in medical discourse, and hence are examples of biologically unproductive females. Even menstruation is then labelled as waste, since it is a wasted ovulatory cycle [10, 11]. Such discourse reduces women to their reproductive potentiality, but also highlights the weight our society still today gives to fertility and reproduction symbolically. Another is that aborted embryos can be said to ‘hover on the borders of selfhood’ ([10, 11], p.74), since they are both associated with a subject, but being dead, also fully is an object. Being an object calls for symbolic classification. It needs to find a place in the world. Since the aborted embryo is also connected as an object with death and decay, it is partly also associated with dirt and waste [8]. It is something that the body of the subject disposes of, such as faeces, urine or in this case; an embryo. Such discharges may trigger disgust, as they are considered unclean. The disgust has many dimensions, but functions primarily to preserve the integrity of the community, as well as of the individual.

### The subject and the abjective

The concept of the abject has previously been employed by Ariss, in order to better understand the cultural role to foetal material [10]. According to Julia Kristeva, the abject is that, which needs to be denied and primally repressed by the subject, in order for it to create and maintain its borders. An abject keeps reminding the subject of its relational dependencies – in the psychoanalytic tradition, primarily to the mother – as well as of its own inevitable death [9]. The abject is to be understood in relation to an object, which is a phenomenon or thing that can (much less dramatically) be comprehended as a fully separate entity, with clear boundaries to the subject. An aborted embryo can then accordingly be understood as abject [9] to a subject, as it has clear connections to an imagined mother, as well as to death.

The researchers we have followed need to provide assurance to society at large and to interested parties, that they treat the ambiguous foetal material properly while making as good use of it as possible. To Douglas,’dirt’ is cultural and leads to pollution behaviour. To medical scientists and other researchers, the potential pollutive properties of embryos are naturals facts. To completely separate these realms would be too naive. Kristeva’s *abject* offers a unifying bridge, since disgust and the abjected protects us from existential anxiety in the face of death or cessation. Medical rituals surrounding the foetus, and other possible pathogenic entities, protect the Self. It does so medically as well as culturally—as they connect these areas to create a meaningful existential framework for individuals as well as communities. The practical as well as symbolic labour that neurobiologists and cell biologists are doing in order to reintegrate foetal material into social and cultural order, have gone more or less unexamined. This article is an ethnographic as well as conceptual contribution to this blind spot.

It is time to move into the premises where the practical refinement of waste to resource actually takes place; the cell and animal transplantation laboratories.

## Results

### Observation of laboratory procedures

When entering the cell laboratory where the tissue starts its transformation, the laboratory personnel need to change clothes and disinfect in an airlock room, before going into the laboratory. In the room there is sharp white lighting from fluorescent tubes, facilitating an easy overview of all surfaces and equipment. The room is quiet and free from odours. There are airflow hoods, microscopes, incubators and cell counting machines, as well as smaller instruments for dissection, as well as pipettes, tubes and disposable gloves in marked drawers and boxes. It is here that Emma and her colleagues process the cells into suspension. The procedure involves receiving an aborted and donated embryo from the nearby abortion clinic; dissecting it, collecting its brain, and then dissociating the cells from the relevant brain region in different solutions, until a so-called cell suspension is what remains. The suspension is a liquid preserving and creating a good environment for the cells it contains. This injection liquid is what will be transplanted to rats or in human patients.

The animal house and laboratories, where the cell suspension is transported when it is not transplanted to human trial subjects, are located behind a totally anonymous door. In the so-called stereotactic laboratory,^4^ researchers are already working on anaesthetized female albino rats in their benches. This is where James and his colleagues transplant the foetal cells to lab rats, whose dopamine production has been impaired to mimic the symptoms of Parkinson’s disease. The room contains three working benches with miniature so-called stereotactic frames for brain transplantation to rodents and fume hoods. The cages have been fitted with wooden chips, water, food and nesting material. The locale lies in relative darkness, except from the small but intense spotlights above the operation tables, focusing both the light and the concentration of the researchers on the rats to be operated on. It is in the premises described above that the major part of the transformation of aborted embryos into an injectable, regenerative cell suspension – as well as the transplantations of it to rat, forming the scientific basis for decisions on subsequent transplantations to human trial subjects – takes place. It is also here that the embryo as we recognize it, disappears from sight, and gradually also from mind.

In the interviews that followed upon observations in the premises described above, James and Emma often spoke of the cells in ways that made visible their different roles and functions. The cells emerged differently, depending on the practical relations between the cells and the researchers, as well as in between researchers of different professions or tasks. The cells were often assigned double roles and characteristics simultaneously in the content and character of conversations and practical situations with colleagues, as described by the participants. This embryonic ambiguity also showed discursively in the interviews themselves, when the participants referred to the embryo or the tissue. Exploring these relations, some distinguishable themes based on the roles of the cells emerged in the analysis of the interview material. Categorizations of these themes will now be discussed in relation to ritual and pollution behaviour, a well as to abjection in the analysis, using interview excerpts. The analysis starts by addressing the foetal cell and the processed suspension mainly as a scientific object and a resource in regenerative medicine.

### Cells of human origin

It is not only the practical refinement of the foetal tissue that transforms it into a possible regenerative treatment. The expressions and concepts used when talking about the foetal material also work their magic in making the material emerge as an appropriate object from which to harness regenerative vitality. To adjust the language internally as well as to an external audience is one way of letting science cleanse the human dirt of the foetal material, thus making it into a neutral scientific object. This linguistic cleansing ritual is something that arguably starts already in the abortion clinic, in which the staff wishes to minimize foetal connotations of a potential baby [12]. In the cell laboratory, the ambiguity of the embryo as a symbol usually works in favour of the researchers taking on the task of refining it. Occasionally these linguistic shifts even reveal the hoped-for development and success of the trial for the researchers.

James: Really, you’d say tissue or something, rather than embryo or, you know… Some people might say like “products of conception”. Or “abortion material” or whatever you know. We usually say “tissue” or “embryo”. […] Today we got […] these two patients who consented, […] and some people will go “okay, well there’s two patients; we’ll have two embryos”… Some people will then, refer to it that way. So that happened today as well, and […] I was like “well no we don’t, because it’s just people who consented, we might not collect anything because, either they’re destroyed or…” you, know…

By conflating the consent to donate with an aborted, available and usable embryo, a wished-for future has been envisioned and evoked with the help of a metaphorical spell whereby the consent is transformed into the embryo. Simultaneously, through the change of a few words, the imagined available embryo has made a cognitive leap in the mind of the researcher, from being a waste product to being a resource. The consent is conflated with the potentiality of the cells, but its origin is thereby also hidden in discourse.

In this way metaphors not only affect our communication but also structure our perception and understanding of events and concepts from the very beginning [23, 24]. In other words, we cannot think without metaphors. The use of foetal tissue in regenerative medicine seems to be defendable by the inbuilt moral argument that ‘saving’, in contrast to ‘wasting’, offers [25]. The connection to another living human being – and the fact that the embryo could potentially have achieved personhood itself – makes it too human to ignore. The metaphorical dichotomy of ‘wasting’ versus ‘saving’ has the power to ease the discomfort that the human origin of the tissue may bring, by at least knowing that it is made use of and not thrown away.

To be capable of being made into a resource to utilize, the embryo needs to be mentally, as well as emotionally and culturally rejected and abjected – in this case by being linguistically substituted for, disguised and hidden in speech. When engaging practically with the material in laboratory, its humanness emerges in other more evident ways, making such mental leaps more far-fetched. Still, the humanness also seems to disappear easily through the same kind of logic, when the human features of the embryo are physically removed in the laboratory.

Emma: That aspect totally disappears.

Andréa: When does that happen, do you think? I mean, in your work?

Emma: When […] the embryo first arrives, then sometimes it occurs to me: “Yeah well, this could potentially develop into something”… but then when you start cutting the head off and take the mesencephalon (the relevant brain region) out… then it is just a piece of tissue that…

Andréa: Yeah, right… (pause) So as soon as you start using your tools… its kind of…?

Emma: Yes, and take away the facial tissue, then it’s not in a way, like, well you don’t think like that anymore…

When Emma does not, literally, need to face the foetus anymore, it becomes an object in her hands. The removal of human features may very well be interpreted in this context as an unmasking of its inner potential, as a source of cells for science and society. This cleansing ritual is meticulously choreographed by standardized guidelines and releases the great hidden vital powers of the embryo. As Douglas argued, it is the identity that makes rubbish dangerous [8]. When marks of origin and identity are removed from the waste, so is the threat it constitutes.

Nowadays James does not have to engage with the dissection of the embryo, as he also did in the past. Now he only receives the ready-made cell suspension for transplantation. When the features making us recognize the embryo as human are already removed, and its format is totally transformed, the situation is different. It has now entered a new phase in the refinement process. It is a new starter product with a new trajectory altogether.

James: But I mean that’s strange in a way because… in what I do now I just get like, a cell preparation. I haven’t seen where it’s come from. It’s very unproblematic for me now. It’s just: “get it, do it”. The same as if I got cells from mouse tissue or rat tissue or from cultured cells, you know, I don’t feel any difference. But that’s when I’ve had no hands in the, the kind of, acquisition of the tissue. […] But I’ve also been exposed to it. If I was told the age (of the embryo) I can in my head imagine what it looks like and…

Andréa: You have a reference?

James: Exactly, yeah, but then I don’t do that (imagine it).

The cell suspension is to the embryo as what the steak in the supermarket is to the cow: refined and cleansed from features showing the origin of the species. In a sense, a reversed ritual logic has been activated. As metaphors or metonyms structure our experiences [23, 24], they are common components in ritual too, helping to enact the wished-for result. Concepts of origin, whole and part play important roles in this process [26]. But rather than admitting and underscoring the relation between embryo and cells, the processes in which the researchers engage work to erase or obscure this relation. However, as we have seen in the quotations, such an endeavour is not easily achieved throughout on all levels. The connection of humanness to the cells they procure is re-enacted on daily basis. It re-emerges in the interactions of the scientists, the connections to aborting women, and in the interactions with the outside society. In the larger community and society outside biomedical research – in which of course the researchers are also members – the cells are usually part of a human embryo, and the embryo has once been connected to a woman.

Whether the cells are expressed as considered part of the embryo, equal to the embryo, or not related whatsoever in the accounts of the researchers, is – as has been repeatedly shown in these accounts – therefore mainly a question of the researchers’ connections to different phases of the process, and their individual relation to and experience of these phases. Still, the cells to some extent resist being easily cleansed of their pollutive properties of sex, death and flesh – as well as the reincorporation into cultural and scientific order.

### The cell as object, subject and abject

To make an aborted embryo into a resource, it first has to be acknowledged as one in culture and society. Labelling the foetal material waste can be said to release the latent ‘biovalue’ [11] of the embryo, and the material becomes an available scientific *object* whose regenerative qualities may be harvested and utilized. Its great therapeutic value in the potential to bring, prolong or enhance the life of another being makes a perfect argument for use in regenerative medicine and neuroscience.

In the following, the researchers talk of their professional tasks mainly in relation to the status of the cells as objects with quality and potentiality. By doing this, they confirm a mutual vision of a resourceful vitality, as well as a common effort to harness it. Here they elaborate on how they view their respective roles and responsibilities within the trial in relation to its goals:

James: I guess my main goal is to ensure that, to kind of to test and validate the quality of that (the cell suspension). So, I think that my main goal has been to, kind of, assess and validate as best we can, the product that then will be put into the holes (in the brain of the rats). You know, it’s the first part of the study.

The foetal material is here seen as a medical object that can be used to produce cell suspension. James argues that it is the quality of the cell that is central, something that is also highlighted by Emma: “It has to be good quality all the way. I mean the embryos need to be of good quality.” The cell quality is of course central to the evaluation and use of all cell kinds in research and trials, but capturing and making use of the potentiality in the standardized way required in evidence-based medicine proves hard when using foetal material. It keeps eluding the researchers time and again, due to its non-standardizable nature. This is something that James works hard for, to have an outcome of good quality: “We don’t know what predicts that so, it’s just more or less seeing how often we can get a good… outcome”.

The practice of preparing and nurturing the foetal cells once in petri dishes or tubes of suspension, may not differ much from work done with neuronal cell types from other sources. Still, the work that needs to be done to foetal cells before reaching that dissolved state; the abortion, the dissection and dissociation – arguably influences the researchers relation to them, as well as the properties of the cells themselves to some degree. As the cells are of abortional origin, their viability seems to be a bit more unpredictable than in other cell types according to the researchers. There seems be two distinct explanations. The first is that a medical abortion procedure—which a significant amount of the foetal material derive from—causes a higher degree of cell death immediately. The second is that the dissociation process from solid material to smaller cell clusters, is a crucial and challenging point where a certain set of skills and sensitivities are required in order to prevent the cells from tangling and sticking in clusters, otherwise rendering them useless [27]. Arguably, this process requires certain competence and care in the work with foetal neural cells, that is not necessarily required in working with e.g. hESC derived from leftover IVF embryos.

Both Emma and James have a clear focus on the work of ensuring the quality of the cells, and they struggle to process them procedurally, as required within evidence-based science. The variability as well as the scarcity of the material makes it difficult. The foetal tissue seems to resist standardization as well as predictability in the hands of science, since it is an organism as much as it is an object. It seems difficult to disarm as a source of polluting uncertainty and symbolic threat. Not only do the cells themselves behave wildly and resist the common task and vision of the trial in becoming pure, harnessed and reusable objects. The researchers themselves sometimes acknowledge the vitality of the cells as a life force in its own right, and as a kind of being or existence to be taken care of; as Emma describes it, the cells “cannot be stressed”. In her practice this is central in how to work with the cells:

Emma: How hard you pipette them and that, it’s just a feel you have for it. […] It’s also something that we don’t have any exact protocol for how to do it. […] I think of them as resting. – That in a way they lie resting there. That they get a chance to just lie there. […] Until it is time… because you don’t want to do something to them, I mean you don’t stimulate… To a certain degree they will probably divide and so on, but since they are small tissue pieces still it will not happen to any great extent. But of course, one can imagine it happening, at least in the beginning… But otherwise, it’s more a matter of trying to imagine them just lying there sleeping.

Imagining the cells as remains of an embryo with the former possibility to develop into a human being may have influenced the way in which the researchers talk of the cells as subjects, as more advanced organisms. Still, there is reason to believe that it is the foetal dissection and tissue cleansing procedures that precede these steps, are what prompts the researchers to talk of the cells as ‘resting’ or ‘sleeping’. In some ways, this discourse recalls the need for sleep in a living infant.

Acknowledging the cells more in terms of subjecthood, the researchers are increasingly reminded of their human origin, as well as of their own deadliness. James highlights this when he says: “to give them an environment that’s protective and allows them to grow. Because if you didn’t do that, they’d kind of be attacked and just die off, so…”.

Modern medical techniques – for example, pre-natal sonograms and diagnosis, foetal surgery, and assisted reproductive techniques including pre-implantation diagnosis – can be said to have pushed the boundaries of personhood, making the subjectivity of the unborn living foetus a philosophical and moral issue of debate (see e.g. [28–30] p.212). But it seems difficult to entirely escape the pollutive fatality of the foetus, even though it is a requirement for the trial to succeed and for cultural reintegration of the aborted embryo to take place.

In this way the cell is not only conceptualized as an object or a subject, but as something in between. Being rejected discharge of sorts, it can be understood as abject [9]. The symbiotic inseparability of the foetus and the mother is, in the case of foetal cells, highlighted by the extra vulnerability these cells have in the laboratory. The cells now need to be cared for by researchers, instead of by the womb of a woman. They are either fully dependent on a researcher’s care, or they are dead. Foetal cells can, in a sense, therefore be said to be concentrate of the process of abjection. They are either dependent or dead, but also the product of a disgusting discharge. The disgust aspect of the foetal material is something that Emma reflects on, in relation to medical sterility and antiseptic measures:

Emma: In the beginning you can feel that is in a way a bit… dirty. The tissue you get… because it has bacteria in it. I mean it came out, through that passage, and it is not really very… there is lot of bacteria and stuff there. You see blood that comes with it and… There is a risk that you could potentially be contaminated yourself too.

The lingering pollutive properties of the aborted embryo are not solely cultural. As a source for a potential transplant in regenerative medicine, it also needs to be controlled for and cleansed from bacteria and potential pathogens. However, not all contagion is biological. There seems to be a migration in meaning concerning the kind of dirt associated with these cells. James talks about this: “I mean I guess, because it’s been treated so much like, washed and dissociated with enzymes and then cleaned again and washed, you know, then it’s, I guess it’s… sterile (laughs)! It should be sterile.” The quotation seems to leave us with a question more than an answer. It reveals a kind of uncertainty as to whether we can trust that the sterilization process has really succeeded on all imaginable levels. And indeed, the connotations that an aborted embryo has seem somewhat hard to wash away, no matter what components are used. This is also something that Emma talks about: “Really, I mean it is tissue that comes from… yeah, vaginally, and there are bacteria and all sorts of stuff. It’s not really very clean. I mean, I feel that stem cells can be controlled in another way.”

It can be argued that there is good reason to be wary of contaminants when working with material that has had so much contact both with the insides of the human female body and with the vaginal canal. However, it was mentioned numerous times during planning group meetings that the serology and pathogen tests looked so very good generally, and that not much contamination except for harmless lactobacillus, normally found on healthy skin, was usually found. Still, that did not seem to take away the fear of the polluting embryo. It is, as discussed in the introduction and in the theoretical section, not only by its biological processes that the foetus or the embryo is a risky object. The dirty properties of the embryo remind us in different ways of fleshy humanness, as well as of an often culturally unsanctioned female sexuality. Even if the women from whom the material is collected may be labelled risky mainly because of possible infectious disease; they may be labelled so *also* by going through an abortion, which is often perceived as connected to sexual behaviour perceived as risky and irresponsible [12, 13]. Here, we want to underscore that the interviewed researchers in no way signalled that this was part of how they view the aborting and donating women. However, it can be argued that such notions strongly still influence the ways in which we all symbolically interpret meaning and value in the world.

## Discussion

This article is an attempt to develop a framework for interpreting the transformative cultural, biological and technical processes involving practically as well as symbolically marginal embryos. Using Douglas’s concept of pollution behaviour combined with Kristeva’s concept of the abjective, we highlight different discursive strategies utilized by the researchers to handle cognitive and emotional challenges posed by the processing of symbolically ambiguous foetal tissue.

Making claims about pollution behaviour and its implications in the processing of foetal cell suspension, relying on a sample of only two interviews, may seem bold. The results should therefore not be considered generalizable to all researchers working with foetal material, but rather illuminate and elaborate on some social and cultural values and meanings that have been connected to the embryo as a symbol [1], and therefore arguably influence the ways in which it may be handled practically. As there are other studies of regenerative use of foetal material in medicine suggesting similar results, it adds weight [10, 12, 13].

We found that the practical, physical removal of the human features of the embryo – as well as the metaphorical and symbolic cleansing in and by language of the foetal material [21, 23–25] – seems to help the researchers escape the uncomfortable and polluting humanness of the embryo, in a variety of practices and situations. It also takes the material a step closer to the pure source of vitality that is the cell suspension. The practical work of refining the tissue aligns with linguistic work [30] in order to disconnect it from all human (and motherly) associations and features, as well as from its connections to decay and death. A secondary effect thereof, is that the subjectivity of the researchers is protected, as the foetal material is abjected [9].

The inherent vitality of the embryo is literally hibernated in time and space and made mobile. The essence of life has eventually been disconnected from death. And, as Douglas notes, death and bodily dissolution is the question to which pollution behaviour and ritual is the answer: “Just as the focus of all pollution symbolism is the body, the final problem to which the perspective of pollution leads is bodily disintegration” [20]. To Kristeva, the answer to this same problem, would be abjection.

The many steps (often back and forth) in between rat models and possible therapeutical application of a clinical trial, cannot be understated. Still, the embryo needs to be abjected also on an organizational level [31], in order for the trial to keep on striving towards the (relatively far-off) common future vision of safe and possibly lifesaving cell transplantations.^5^ This abjection is partly achieved by the neutralizing and technical language in protocols and documents guiding the practice, as well as by organising the trial in a manner so that minimal ethical issues arise [27].

However, the accomplishment of disjointing life from death has not come easy in practice, as has been shown. It takes many different types of labour to make science and culture out of nature. The scientists in the cell and animal transplantation laboratories have to master all of this when working hard to tame the unruly foetal cells. They must manage the materiality of the cells and also, as has been shown here, their linguistic treatment. The researchers take on the task of refining the aborted embryo from trash to treasure. They may in return enjoy a position in which their otherwise questionable closeness to the dirty embryo will not be questioned. They are protected from pollution by the abject [9], by momentarily jeopardizing their own cleanness to restore social and cultural order.

Rituals involving elements of collective aggression and sacrifice are believed to help create a sense of community. The irreversibility of the sacrifice transforms its participants [26]. How may we then understand the sacrifice of the embryo to science? Imposing death on it by abortion seems to protect the scientists from being able to commit any further violence against it. All subsequent handling of the foetal material – no matter what the character – is then understood rather as an act of saving its remaining vital properties. Practically dismembering the embryo under microscope and diluting certain brain cells of it into a suspension would under different circumstances be considered an act of violence. However, when narrated as part of a cleansing process, it has a higher purpose, which is to make trash into treasure and thus protect the community from pollution. The scientists are then – together with the embryos they handle – as professionals, always in a liminal,^6^ intermediate, state. They are not only allowed but also encouraged to commit deeds, which would in other circumstances have been deemed pollutive. Acting under this circumstantial autonomy enables them to take on the burden of responsibility for cleansing the embryo of symbolic dirt. Scientific practice legitimately restores the order of the community, by cleaning the foetal material from its associations with dirt, pollution and liminal humanness.

## Conclusions

It is crucial to understand the rituality and cultural concern surrounding the handling of foetal material in regenerative research. By applying Douglas’s concept of pollution behaviour [8] to the aborted embryo, and by understanding it as abject [9], we found that the tabooing of it activates an adapted and sensitized language. The linguistic substitutions, ascribing different properties to the cells depending on context and purpose, are indeed connected to the knowledge production of the trials. They also help the escaping from the decaying fleshiness of the embryo.

The substitutions enable the use and refinement of a tissue around which there is practical consensus but cultural ambiguity. This may, however, complicate communication and cognitive and emotional processing amongst the involved researchers, due to lack of common definitions. Cultural ambiguities will, on the other hand, always exist and do important developmental value work in communities and societies. The taboo [8] of the embryo helps protect a certain practical core consensus of the research community concerning the rituals involving the cells. This means that, even if there is no consensus about what the cells are, there is consensus about what the cells can do, and what can be done to the cells. Understanding the ambiguous cultural symbols of a community is as important to science as it is to religion and society. It may help us see the interconnectedness between these seemingly separate areas. There is much to suggest that ritualistic pollution behaviour may be used to fully make sense of a situation where no traditional scientific means of knowing can adequately be applied. It may complement other, more scientifically legitimate ways of knowing. In this study, we argue that the ‘embryonic ambiguity’ of the cells, offers the scientists engaging with the foetal material a liminal cultural key role, when transforming the material from trash into treasure. This is an important insight into the diverse set of skills employed by biomedical scientists. It is also a valuable contribution to previous humanist research on cultural and ethical aspects of the use of aborted embryos and foetal material in regenerative medicine (cf. [11, 12, 28]), as our study focuses on the researchers receiving and processing the tissue, rather than on the parties involved in the donation process [13].

When foetal material brings questions of origin and humanness, of being and non-being, of part and whole and of independency and dependency, as well as of life and death; pollution behaviour offers answers, which the traditional methods employed in the cell laboratories cannot. By neutralizing perceived threats to communities as well as to subjectivities, it also enables progress, development and change. Pollution behaviour may add different types of meaning and understanding also to the laboratories of regenerative medicine, and a broadened framework of action for facilitating the cultural reintegration of abject [9] tissues.

## Data Availability

Data and materials will be archived and made available through Lund University and Arkivcentrum Syd, after Wiszmeg finishes working with them.

## Footnotes

1 The term *foetal* will be employed throughout the article when referring to the procured tissue and the cells, as well as to the processed cell suspension itself. The term *embryo* will however be used when referring to the donated abortion material as a whole, as it best describes the developmental stage of the donations. Even though the trial in the study mainly uses tissue from donations developmentally defined as embryos, their homepage defines the cells used as foetal. Therefore that terminology is adopted when referring to the processed tissue, the cells or the resulting suspension. This choice of term also minimizes possible confusion with human embryonic stem cells. The term embryo refers to the time period of development when organs are formed. In the human, it is defined as up to 7 weeks + 6 days post-conception. The foetal period starts from 8 weeks + 0 days post-conception. It should be noted that the trial occasionally procures and processes cells from what are by definition early aborted foetuses, up until 10 weeks post-conception. This is because measurements and calibrations sometimes are uncertain concerning the exact gestational age. Therefore all donations within a period of 5–9 weeks post-conception may be used in the trial. The tissue may sometimes by definition subsequently be derived from tissue within the foetal developmental stage. Up until this point in development, the embryo does not have all the bodily organs formed, nor functioning. No circulation system has developed yet and the cells survive in part independently of microcirculation.

2 Internal, unofficial documents.

3 See Swedish Transplantation Act 1995:831, and the bylaw SOSSF 2009:30.

4 Stereotactic surgery is a minimally invasive kind of surgery in which a three-dimensional coordinate system is used for locating the sites of action in the body when planning and carrying out an operation.

5 Here, it should be noted that the use of foetal cells on a larger therapeutical scale has never been an option realistically considered, due to ethical concerns as well as scarcity of the material. However, the research on foetal cells is used to develop a template for further research on transplantation with e.g. hESC or iPSC cells.

6 The liminal state of professional scientists should be understood in relation to their private self, and as functioning to ultimately reaffirm cultural social order, even if they temporarily disturb it. See e.g. the anthropologist Victor Turner’s *The ritual process, structure and anti-structure,* 1969.
